# Primary sclerosing cholangitis (PSC) and inflammatory bowel disease (IBD): a condition exemplifying the crosstalk of the gut–liver axis

**DOI:** 10.1038/s12276-023-01042-9

**Published:** 2023-07-18

**Authors:** You Sun Kim, Edward H. Hurley, Yoojeong Park, Sungjin Ko

**Affiliations:** 1 Department of Pathology, University of Pittsburgh School of Medicine, Pittsburgh, PA USA; 2 Department of Pediatrics, University of Pittsburgh School of Medicine, Pittsburgh, PA USA; 3 Pittsburgh Liver Research Center, University of Pittsburgh School of Medicine, Pittsburgh, PA USA

**Keywords:** Translational research, Clinical genetics

## Abstract

The close relationship between primary sclerosing cholangitis (PSC) and inflammatory bowel disease (IBD) provides a good opportunity to comprehend the gut–liver axis. The gut and the liver have reciprocal interactions, including how gut inflammation influences the liver through immune cells and the microbiota and how the microbiota in the gut modifies bile acids, which are produced and secreted from the liver. PSC-IBD shows distinct clinical findings from classical IBD. In addition, a distinct genetic predisposition and unique microbiota composition suggest that PSC-IBD is an independent disease entity. Understanding the pathogenesis of PSC-IBD helps to develop novel and effective therapeutic agents. Given the high risk of malignancies associated with PSC-IBD, it is critical to identify patients at high risk and implement appropriate surveillance and monitoring strategies. In this review, we provide an overview of PSC-IBD, which exemplifies the gut–liver axis.

## Gut-liver interaction: Inflammatory bowel disease and liver disease a distinct condition

Inflammatory bowel disease (IBD) combined with a rare liver disorder should be classed as a separate health condition, with researchers in the USA calling for extensive research. Patients with IBD and primary sclerosing cholangitis (PSC), a severe liver condition involving the inflammation, scarring and eventual blocking of bile ducts, frequently suffer serious complications, including cancer. However, the pathogenesis of PSC-IBD is unclear. Sungjin Ko and colleagues at the University of Pittsburgh, USA, reviewed PSC-IBD and point out that the condition has clinical characteristics distinguishing it from IBD. PSC-IBD involves disruption to both innate and adaptive immunity, and genetic and environmental components contribute to disease risk. Further research is needed into the extensive interactions between gut microbiota and liver bile acids. PSC-IBD patients should be screened regularly to detect associated cancers early.

## Introduction

Since microbiomes were introduced in the biomedical field^1^, the gut–liver axis has emerged as a major field of biomedical research that has found strong bidirectional interaction and crosstalk between the intestine and the liver. The gut–liver axis plays a significant role not only in physiologic conditions but also in pathologic conditions, including noncommunicable diseases and diverse cancers^2,3^. The liver produces diverse biologic mediators, including bile acids (BAs), which are secreted into the intestine through the biliary tract. BAs are especially important for the digestion of fats in the gut as well as for carrying toxins/other substances away from the liver, where they can be eliminated from the body in feces. In the gut, the microbiota metabolizes BAs and other dietary nutrients, which flow back to the liver via the portal vein^4^. In cases of dysbiosis resulting from diverse intestinal disorders, chronic inflammation and oxidative stress can build up and lead to augmented production of endotoxin, dysregulation of BAs and disruption of the intestinal barrier, all of which can impact the liver^5^. For example, compromising the gut integrity can cause translocation of bacteria as well as harmful bacterial products such as lipopolysaccharide (LPS) into the circulation, which can easily reach the liver to produce hepatic inflammation^5^.

An example of this bidirectional gut–liver axis is epitomized by the case of primary sclerosing cholangitis (PSC) and inflammatory bowel disease (IBD). PSC and IBD are immune-mediated diseases that are strongly intertwined. Patients suffering from both conditions, PSC-IBD patients, often experience deterioration of the disease course, which can result in malignancies in the liver or intestine. In this review, we summarize the current understanding of the pathogenesis and clinical characteristics for patients with PSC-IBD, particularly from the gut–liver axis perspective.

### IBD and PSC

IBD is a chronic idiopathic and progressive disorder of the gastrointestinal (GI) tract composed of two distinct classifications: ulcerative colitis (UC) and Crohn’s disease (CD). The incidence and prevalence of IBD is increasing globally, specifically in Asian countries, and is causing a huge socioeconomic burden to their societies^6–9^. The pathogenesis of IBD is postulated to arise from a combination of factors, including genetic susceptibility, dysregulated immune response, impaired intestinal mucosal barrier system, and environmental factors, such as diet and the microbiota^10–12^. During their lifetime, ~50% of patients with IBD develop extraintestinal manifestations (EIMs), which affect the skin, joints, eyes, or hepatobiliary system^13,14^. EIMs of IBD are characterized by lymphocyte infiltration of the affected organs. In particular, hepatobiliary manifestations such as PSC are the most troublesome and can be life-threatening situations. PSC is a rare, chronic, and progressive cholestatic and inflammatory disease that is characterized by intrahepatic and extrahepatic biliary strictures^15,16^. The natural progression of PSC is slow but eventually results in end-stage liver disease requiring liver transplantation (TPL) 15-20 years after PSC diagnosis^17^. Interestingly, this rare disease has a strong association with IBD. Up to 85% of patients with PSC suffer from IBD with a strong predominance of UC over CD. However, only 1–8% of IBD patients develop PSC^16,18,19^.

**Fig. 1:**
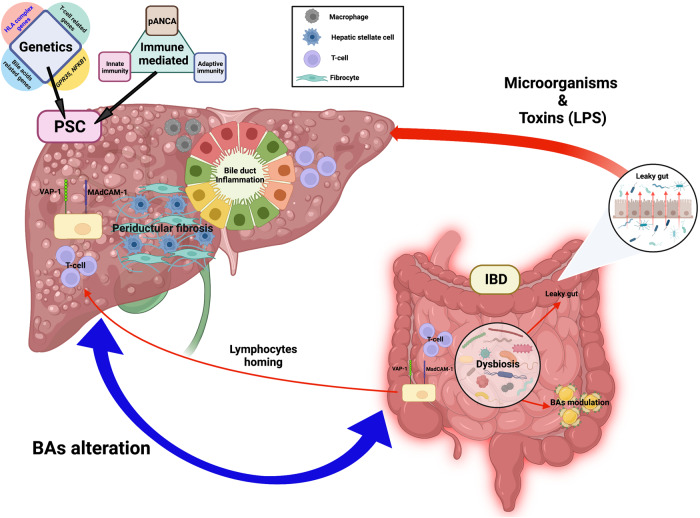
The pathogenesis of PSC and IBD illustrating the gut–liver axis. In the liver, genetic predisposition and immune-mediated pathways are primarily involved in the pathogenesis of PSC with impaired bile acid (BA) homeostasis. Alterations in BAs directly impact the microbiota, leading to dysbiosis and increased translocation of microorganisms and toxins through a debilitated intestinal barrier or “leaky gut”. In turn, a leaky gut leads to bile duct and hepatic inflammation, and dysbiosis further contributes to hepatic inflammation through BAs alterations. Activated gut lymphocytes migrate to the liver through the portal vein and eventually impact hepatic inflammation. Crosstalk between cholangiocytes, immune cells, hepatic stellate cells and myofibroblasts ultimately results in periductular fibrosis, which is the pathognomic sign of PSC. (Generated with BioRender.com). BAs bile acids, HSC hepatic stellate cells, IBD inflammatory bowel disease, MAdCAM-1 mucosal addressin cellular adhesion molecule-1, pANCA perinuclear antineutrophil cytoplasmic antibodies, PSC primary sclerosing cholangitis, VAP-1 vascular adhesion protein-1.

While the pathogenesis of PSC-IBD remains unknown, recent studies suggest that multiple factors, including genetic susceptibility, the immune-mediated pathway, alterations in BAs, and gut dysbiosis, may contribute to the pathogenesis of PSC-IBD (Fig. 1)^20^. In addition, the clinical features of PSC-IBD patients are largely distinct from those of IBD-only patients, suggesting that PSC-IBD is a unique disease entity^21^. Furthermore, the close link between IBD and PSC likely represents an important example of how dysfunction of the gut–liver axis can lead to clinical disease.

### Pathogenesis of PSC-IBD

#### Genetic susceptibility

Despite the increasing incidence of PSC, it remains a rare disease with a pooled incidence rate of ~0.77 (0.45–1.09) per 100,000 person-years in North American and European countries^17,18,22^. The incidence rate of PSC seems to be much lower in Asian^23–27^ and Southern European countries^28^ and seems to have a weaker association with IBD. In Asia, retrospective database studies have found incidence rates of PSC among IBD patients ranging from 0.39% in India^26^, 1.1% in Korea^25^, and 1.57% in Taiwan^29^. These results are substantially lower than those of Western countries, which have reported that up to 8% of IBD patients are diagnosed with PSC^19^. This discrepancy may be attributed to the different genetic backgrounds of IBD between Asian and Western countries^30^. Recent genome-wide association studies (GWAS) found more than 200 susceptible genetic loci for IBD^31,32^, many of which mapped to other immune-mediated disorders, such as psoriasis and ankylosing spondylitis. The low prevalence of PSC made it challenging to identify its predominant genetic background. The national Swedish PSC cohort study (*N* = 678) revealed that the risk of PSC was significantly increased in first-degree relatives, that is, offspring (hazard ratio (HR) 11.5) and siblings (HR 11.1)^33^, implying a genetic component to the pathogenesis of PSC.

The most predominant genetic findings associated with PSC have been linked to the human leukocyte antigen (HLA) complex on chromosome 6 (odds ratio (OR) 3-5)^34,35^. HLAs are proteins expressed on the surface with antigenic peptides involved in antigen recognition by the T-cell receptor (TCR) on CD4 and CD8 T cells. Class ǀ is expressed in all cells, and class ǁ is expressed in antigen-presenting cells such as macrophages^36^. Genetic studies have revealed that the predisposing HLA alleles or haplotypes for PSC include HLA-DRB1*03, HLA-DRB1*13, HLA-B*08:01, HLA-DQB1:02, and HLA-DQA1*05:01, while the protective alleles include HLA-DRB1*04 and HLA-DQB1*03:02^34,35,37,38^. The strong association between HLA and PSC suggests that adaptive immunity may play a crucial role in PSC development. Despite the identification of PSC-specific TCR clones from PSC liver tissue^39^, PSC-specific biomarkers such as antigenic peptides are largely elusive in the clinic.

A recent GWAS analyzing large PSC cohorts advanced our understanding of the genetic background of PSC. More than 20 non-HLA susceptibility loci were found to be significantly associated with PSC^34,35,40,41^. Notably, these genetic foci are strongly correlated with one or more other immune-mediated disorders, such as ankylosing spondylitis, CD, and psoriasis^41^, indicating that PSC is positioned as an immune-mediated or autoimmune disease. In summary, the genetic predisposition for PSC seems to involve HLA genes and genes related to T cells (*IL2/IL21, IL2RA, CTAL4/CD28*, etc.)^35,40–42^, BA homeostasis (*TGR5, HDAC7, etc*.)^35,43,44^, and other inflammatory conditions (*GPR35, NFKB1*, etc.)^34,35,41,45^.

However, PSC shows limited genetic links with IBD^40,46^, despite their close associations. Indeed, UC and CD showed good genetic correlation to each other (genome-wide genetic correlation (r(G) 0.56). Nonetheless, PSC had a significantly lower genetic correlation with UC (r(G) 0.29) and CD (r(G) 0.04) in comparison to that between UC and CD^46^. Strong comorbidity of PSC-IBD is likely the consequence of a distinctive disease entity, which is genetically distinct from classical IBD phenotypes^41^. However, PSC is a complex immune-mediated disease in which the genetic contribution is limited, likely <10% of disease development, suggesting that other environmental risk factors may have a more critical role than genetics^35,41^.

#### Immune-mediated pathway

PSC is an immune‑mediated disease, and many associated risk genes are related to the immune system, although genetic influences on PSC development are limited. The pathogenesis of PSC-IBD involves the perturbation of both innate and adaptive immunity. Several studies have demonstrated abnormal innate immunity in PSC^47,48^, but no unique PSC-specific features have been identified.

Cholangiocytes (bile duct epithelial cells) are essential for the maintenance of mucosal homeostasis in the biliary tract. Cholangiocytes are exceptionally sensitive to systemic immune conditions and have an active role in cultivating the proinflammatory and profibrotic response. Bone marrow cell injection into healthy mice is sufficient to induce pathologic reactivation of cholangiocytes associated with macrophage infiltration^49^, which is a hallmark of cholestatic liver disease including PSC, highlighting the immune-dependent connection between PSC and IBD. Cholangiocytes possess an innate immune system consisting of Toll-like receptors (TLRs) and constantly interact with pathogen-associated molecular patterns (PAMPs, including LPS and other antigens), which circulate from the gut^50^. Under physiologic conditions, cholangiocytes can maintain homeostasis through tolerance to these antigens. However, a diminished tolerance to antigens and dysregulated biliary innate immunity are found in cholangiopathy^47,50^. Moreover, cholangiocytes produce and secrete proinflammatory and chemotactic cytokines such as interleukin (IL)-1α, IL-6, and monocyte chemotactic protein-1 (MCP-1/CCL2) through dysregulated innate immunity^51^. Given the strong link between PSC and IBD, cholangiocytes in PSC may be exposed to an increased amount of LPS or other antigens released from the inflamed gut, which then triggers TLR-mediated signaling. Indeed, primary cholangiocytes isolated from PSC patients exhibited increased TLR protein expression and activation of the myeloid differentiation factor 88 (MyD88) signaling complex, resulting in aberrant innate immune activation with an absence of tolerance^47^.

Cystic fibrosis transmembrane conductance regulator (CFTR) is selectively expressed by cholangiocytes, where it regulates bicarbonate secretion. Defective function of CFTR impacts the innate immunity of cholangiocytes and is associated with cholangiopathy^52^. In dextran sodium sulfate (DSS)-induced colitis of CFTR knockout mice, challenge with LPS evoked biliary damage and portal inflammation, suggesting that translocated LPS impacts the polarity of cholangiocytes to generate a strong TLR4-mediated inflammatory response^48^. Thus, intestinal inflammation causes increased leakage of LPS and may cause biliary damage in a genetically susceptible host through the LPS–TLR4–NF-κB axis pathway^53^.

Adaptive immunity associated with HLA genetics also plays a pivotal role in PSC pathogenesis. Similar to celiac disease, which involves continuous adaptive immune activation to gluten, the continuous HLA-antigen-T-cell interactions evoked by exogenous and endogenous antigens could be implicated in PSC development^54^. In fact, hepatic T-cell infiltration in PSC patients is evident, as is the case in other tissues of EIMs associated with IBD^39^. T helper (Th)17 cells are key players in the defense against bacteria and fungi, are involved in autoimmune responses^55,56^, and have been found to be increased in PSC patient responses to pathogen stimulation. Moreover, IL-17A-expressing lymphocytes were frequently observed within the periportal area of PSC livers^57^.

Additionally, several studies have suggested that the IL-2 signaling pathway plays a role in PSC development^40,42^. Interestingly, mice with genetic deletion of IL-2 receptor subunit alpha (IL2RA, CD25), a representative marker for regulatory T (Treg) cells, spontaneously develop periportal inflammation with biliary duct injury and colitis mimicking human IBD^58^. Abundant T-cell infiltration in both the liver and colon was observed, indicating the significance of adaptive immunity in the pathogenesis of PSC-IBD^58^.

Several autoantibodies have been reported in patients with PSC, including perinuclear antineutrophil cytoplasmic antibodies (pANCAs), anti-biliary epithelial cells, and antinuclear antibodies. The existence of autoantibodies in PSC reflects abnormal immune responses and is considered evidence of immune-mediated disease^59^. pANCA is a serologic marker of UC, and the presence of pANCA in PSC may reflect the overlapping pathway toward gut antigens. pANCA positivity in PSC patients is variable, ranging from 39% to 70%^60,61^. pANCA positivity is higher in extensive UC patients with subclinical PSC than in those without subclinical PSC^62^. Furthermore, HLA-DRB1*03 was more prevalent in pANCA-positive UC patients than in pANCA-negative UC patients^60^.

#### Gut-lymphocyte homing hypothesis

The strong linkage between PSC and IBD implies hepatic inflammation originating in the intestine. The gut barrier is composed of tightly bound enterocytes with apical junctional proteins that restrict the passage of microbes and other antigens from the gut lumen^63^. Nevertheless, during gut inflammation, barrier integrity is impaired, and permeability is increased^64,65^. This condition is referred to as the “leaky gut”, and microbes and other microbe-derived proinflammatory molecules can transverse from the gut lumen to the liver through the portal system, leading to hepatic inflammation.

However, hepatic inflammation can be caused independently from gut inflammation. Indeed, PSC can develop many years after proctocolectomy in the absence of gut inflammation^66^. In this case, several studies have suggested the role of gut-homing lymphocytes. During active gut inflammation, effector T cells develop and persist as long-lived memory cells^67^. Stimulated T cells can enter the liver through the enterohepatic circulation. Gut adhesion molecules consist of chemokine ligand 25 (CCL25) and mucosal addressin cellular adhesion molecule 1 (MAdCAM-1). Under physiological conditions, the expression of CCL25 and MAdCAM-1 is restricted to gut-associated lymphoid tissues. When gut inflammation is triggered, the expression of gut adhesion molecules is increased, and the recruitment of T cells to the gut is also enhanced via gut-homing receptors of C-C chemokine receptor 9 (CCR 9) and alpha 4 beta 7 (α4β7) integrin^68^.

Interestingly, the expression of MAdCAM-1 and vascular adhesion protein 1 (VAP-1) along with CCL25 secretion are shared in the digestive and hepatic systems of PSC patients, which enables activated T cells to bind to both the intestinal mucosa and hepatic endothelium^67^. The portal infiltrates in PSC patients showed mainly long-lived memory T cells originating from the gut, with a considerable proportion of them expressing the gut-homing integrin α4β7 and/or CCR 9^39^. Thus, gut memory cells can be activated within the liver, leading to persistent hepatic inflammation even after gut inflammation subsides. Dual-homing lymphocytes may explain how hepatic inflammation can remain independent of gut inflammation.

#### Impaired bile acid homeostasis

Cholestatic liver disease, including PSC, is a chronic condition that is characterized by reduced biologically toxic bile flow and consequent impaired systemic BA homeostasis^69^. BAs are important regulators of metabolism and the immune system. The synthesis, secretion and/or metabolism of BAs are strictly regulated to maintain BA homeostasis. Cholangiocytes are continuously exposed to high concentrations of BAs, so they produce a “bicarbonate umbrella” to protect themselves from BA toxicity^70^. Primary BAs are synthesized in the liver from cholesterol and excreted into the small intestine. Approximately 95% of BAs are reabsorbed in the terminal ileum by the apical sodium-dependent bile acid transporter (ASBT), a protein located in the enterocyte, and enter the enterohepatic circulation, a major component of the gut–liver axis^71^. This well-organized process maintains systemic BA homeostasis. However, conjugated and unconjugated primary BAs were elevated in PSC patients, indicating impaired BA homeostasis^72,73^.

The signaling of BAs involves a series of receptors, including membrane-bound G protein-coupled receptor (TGR5) and the nuclear receptor farnesoid X receptor (FXR)^43,74,75^. BAs act as ligands for both FXR and TGR5 and are important regulators of BA homeostasis as well as the regulation of immunity. TGR5 is colocalized with CFTR in the apical membrane of cholangiocytes and is considered to be involved in modulation of the bicarbonate umbrella^34^. Deficient bicarbonate secretion in PSC patients might be associated with downregulation of TGR5 in cholangiocytes^35,76^. Resequencing of TGR5 revealed a strong association between the TGR5 single-nucleotide polymorphism rs11554825 and PSC (OR 1.14) and UC (OR 1.19), suggesting the involvement of TGR5 in the pathogenesis of PSC-IBD^43^.

FXR is a key signaling pathway in BA homeostasis that directly regulates BA synthesis and is also involved in immune modulation, as well as in maintaining intestinal epithelial barrier function. BA synthesis is regulated by both intestinal and hepatic negative feedback through the enterohepatic circulation; activation of ileal FXR by BAs stimulates the synthesis of fibroblast growth factor 19 (FGF19), which regulates hepatic synthesis of BAs. In hepatocytes, FXR triggers an FGF receptor 4-dependent signaling pathway and suppresses BA synthesis^71^. The accumulation of BAs in the livers of PSC patients is frequently associated with aberrant hepatic FGF19 expression^77^. In addition, other receptors or transporters also play a role in BA homeostasis, including anion exchanger 2 (AE2) and ASBT. AE2 is located in the cell membrane of cholangiocytes, and downregulation of AE2 was reported in PSC-UC patients who showed increased BA toxicity^78^.

Primary BAs are converted to bioactive molecules by enzymes of the gut microbiota, and bioactive metabolites serve as substrates for microbiota metabolism, modulate the balance of Th17 and Tregs, and inhibit bacterial growth^5,71,79,80^. Therefore, there is reciprocal action between BA metabolism and the gut microbiota. Numerous studies have suggested that intestinal inflammation and the resulting gut dysbiosis may influence BA homeostasis^71,81^. A preliminary study was conducted to investigate the mucosal microbiome, gene expression and cellular immunity between PSC-IBD patients (*N* = 10) and UC-only patients (*N* = 10). Significant disruption of BA signaling pathways was observed in PSC-IBD patients compared to UC-only patients (*P* = 0.02), indicating the involvement of the perturbation of BA homeostasis in the PSC-IBD pathogenesis^82^.

#### Fibrosis

The pathognomic finding of PSC, that is, “onion skin” scars, appears as peribiliary circumferential fibrosis layers, causing fibrous obliteration of the bile duct. Although cholangiocytes are generally resilient to BA toxicity, prolonged exposure to BAs can cause chronic senescence^83^. Senescent cholangiocytes make the surrounding tissues to the senescence-associated secretory phenotype and resistance to apoptosis, resulting in persistent inflammatory and fibrosis responses^84^. In PSC patients, cholangiocytes are activated by BAs, proinflammatory cytokines and the gut microbiota^85^. Reactivated cholangiocytes interact with myofibroblasts, immune cells and hepatic stellate cells (HSCs) in the process of peribiliary fibrosis^86,87^. The details of the cellular communication between cholangiocytes and other cells remain elusive, necessitating future studies using advanced techniques such as single-cell transcriptome analysis.

#### Gut microbiota dysbiosis

The close relationship between IBD and PSC has led to the involvement of the gut microbiota as a major contributing factor to the development of PSC. Dysbiosis in IBD patients is evident in that a decrease in the bacterial diversity and an increase in the composition of certain virulent bacteria could exaggerate the immune response^88,89^. Moreover, the features of the microbiota in PSC patients are distinct from those in healthy individuals^5^.

Importantly, the gut microbiota in PSC patients has been found to be functionally different than that in healthy controls, exhibiting a decreased level of vitamin B6 and branched-chain amino acids (*P* < 0.0001)^90^. Furthermore, the dysbiosis in PSC patients was distinct from that in IBD-only patients, characterized by a significant overrepresentation of the *Enterococcus*, *Fusobacterium* and *Lactobacillus* genera^91^. In a substantial comparison of the bacterial DNA between PSC-IBD patients (*N* = 85), healthy controls (*N* = 263), and UC-only patients (*N* = 36), reduced bacterial diversity and a different global microbial composition were observed specifically in PSC-IBD patients compared with healthy controls (*P* < 0.001) and UC-only patients (*P* < 0.01)^92^. The abundance of the *Veillonella* genus showed a marked increase in PSC-IBD patients^92^. Another study also described reduced bacterial diversity and significant alterations in microbial features in PSC-IBD patients compared to UC-only patients^93^. *Rothia*, *Enterococcus* and *Streptococcus* were more abundant in PSC-IBD patients than in UC-only patients^94^, implying unique crosstalk and the impact of the gut–liver axis in the case of the PSC-IBD combination.

Gut inflammation may lead to subsequent hepatic inflammation. The role of commensal microbiota in biliary damage was evaluated using the NOD.c3c4 mouse model. In germ-free circumstances, NOD.c3c4 mice exhibited less damage to bile ducts, suggesting a role of the gut microbiota in biliary damage^95^. However, another study demonstrated the opposite results: germ-free multidrug resistance 2 (MDR2, encodes a biliary transport protein) knockout mice showed severe biliary damage, suggesting that the commensal microbiota has a protective role in murine cholestasis^96^. Together, dysbiosis in PSC may trigger context-dependent effects on biliary injuries. For example, *Klebsiella pneumonia* may disrupt the intestinal epithelial barrier and initiate bacterial translocation. Fecal microbiota from PSC-IBD patients were transferred to gnotobiotic mice and caused activation of Th17 cells in the liver of the mice^97^. Bacterial culture of mesenteric lymph nodes of mice isolated *K. pneumoniae, Proteus mirabilis* and *Enterococcus gallinarum*, which were frequently detected in PSC patients^97^. Therefore, *K. pneumoniae* may induce hepatobiliary tract damage secondary to inducing the hepatic Th17 cell-mediated immune response^97^. These hepatobiliary tract injuries can be reduced after antibiotic treatment, indicating the role of the pathogenic microbiome in intestinal barrier dysfunction and liver inflammation^98^. Another study demonstrated that enrichment of *Lactobacillus gasseri* in MDR2 knockout mice causes disruption of the gut barrier. *L. gasseri* easily translocates to the liver, activates T cells, and increases the production of the proinflammatory cytokine IL-17^99^. The IL-17-mediated liver inflammation and fibrosis are noted as being similar to those in PSC. Alterations in the gut microbiota in PSC might be a secondary event in the disease, whereas the gut microbiota is also involved and has an active part in disease progression through aspects involving gut barrier dysfunction and the translocation of virulent bacteria and toxins. Additionally, given that the intestinal dysbiosis of PSC is independent of IBD, the gut microbiota may play a pivotal role in the pathogenesis of PSC^5,91,98^; thus, comprehensive examination of gut–liver interactions using diverse preclinical cholestasis models in combination with IBD features will be fundamental.

### Clinical characteristics of PSC-IBD

PSC is usually asymptomatic in the early stage; however, as the disease progresses, patients complain of nonspecific symptoms such as fatigue, pruritus, fever, and weight loss. In advanced cases, abdominal pain and jaundice often develop. In IBD patients, PSC can be diagnosed when biochemical screening reveals abnormal liver chemistry findings, especially serum alkaline phosphatase (ALP) levels. In patients with PSC-IBD, IBD may manifest prior to or be concurrently diagnosed with PSC, while in some cases, IBD becomes apparent at a late stage of PSC, even after liver TPL. In a retrospective cohort study, of 84 PSC patients with no evidence of IBD at the time of liver TPL, 22 (26.2%) patients developed IBD after liver TPL^100^.

Due to subclinical symptoms or the absence of biochemical findings that characterize PSC in the early stages, PSC may be underdiagnosed in IBD patients. In fact, using magnetic resonance cholangiography (MRC) analysis of long-standing IBD patients (*N* = 322), 24 patients (7.5%) were found to have PSC, which is ~3-fold higher than the 7 patients (2.2%) diagnosed with PSC based on symptoms^101^. PSC-IBD patients have distinct clinical features compared to conventional IBD patients. PSC-IBD patients typically show a male predominance and more commonly features of extensive colitis, rectal sparing and backwash ileitis than IBD-only patients^15,26,29,102–104^. Additionally, a predominance of right colon inflammation is observed^105^.

Progressive PSC patients requiring liver TPL tend to have a milder course of IBD compared to the no liver TPL group, as evidenced by the lower use of steroids (*P* = 0.025) and lower surgery rates (*P* = 0.006), suggesting that the severity of PSC may have a protective role in intestinal inflammation^106^. Approximately 30% of PSC-IBD patients who received liver TPL showed active disease activity and poor clinical outcomes of IBD^16,107^.

A Swiss IBD cohort study identified risk factors for the coexistence of PSC in patients with UC, such as nonsmoker at diagnosis (OR 9.253, *P* = 0.030), history of appendicectomy (OR 4.114, *P* = 0.019), male sex (OR 2.771, *P* = 0.022), and extensive colitis (OR 2.855, *P* = 0.011)^108^. The Mayo PSC risk score based on age, bilirubin, serum AST, albumin and history of variceal bleeding is a valid prognostic parameter for PSC patients^109^. A recent study revealed poor prognostic factors of PSC, including male sex, the presence of dominant strictures, coexistence with IBD and high serum ALP levels^110^. Importantly, the overall survival rate of PSC-IBD patients was significantly lower than that of IBD-only patients (*P* = 0.001)^108^.

### PSC-IBD and colorectal cancer

IBD is an important etiology for colorectal cancer (CRC) development, and the extent of colitis and a long duration of disease are the major risk factors associated with CRC^111^. Remarkably, the risk of CRC in PSC-IBD patients is significantly higher than that in the IBD-alone cohort. In a meta-analysis evaluating the risk of CRC and colorectal dysplasia in PSC-UC patients, an increased prevalence was observed, with ORs of 4.79 and 5.11, respectively, compared to UC-alone patients^112^. A population-based cohort study demonstrated that PSC-IBD patients had an increased risk of CRC (HR 2.43, *P* < 0.001), were more likely to be right colon dominant and presented at a lower median age (59 year vs. 69 year, *P* < 0.001) compared to IBD-only patients^113^. Another cohort study also reported a higher CRC incidence in IBD patients when PSC was detected (3.3 cases per 1000 patient-years)^114^.

Taken together, combined PSC is a convincing risk factor for developing CRC in IBD patients, although the mechanism by which coexisting PSC increases the risk of CRC remains largely unknown. However, the carcinogenetic effects of BAs on CRC development have emerged and involve FXR and TGR5^71^. Furthermore, the risk of CRC development in PSC-IBD patients is persistent after liver TPL, and the cumulative risk of CRC after liver TPL was 0.6%, 1.8%, and 3.3% after 5, 10, and 20 years, respectively^115^.

Therefore, strict surveillance of annual colonoscopy from the time of diagnosis of PSC-IBD, even after liver TPL, is strongly recommended for early detection of CRC^116^. The chemopreventive effects of UDCA on CRC development in PSC-IBD patients are controversial. A meta-analysis of PSC-IBD patients (*N* = 763) showed no significant protective effects of UDCA use on the development of colorectal neoplasms (OR 0.81)^117^. Current guidelines demonstrate that UDCA is not recommended for the prevention of CRC in PSC patients^116^.

### PSC-IBD and cholangiocarcinoma

Cholangiocarcinoma (CCA) is the second most common liver cancer and has steadily risen over the past two decades^118^. Approximately 15% of PSC patients are reported to develop intrahepatic CCA with an OR of 22.9, as determined by a meta-analysis^119^. Thus, one of the most dreaded complications of PSC-IBD is the development of intestinal and hepatobiliary malignancies, notably CCA, which has a <10% 5-year survival rate. Often, the early symptoms of CCA, such as weight loss, jaundice or abdominal pain, are also seen in PSC and do not necessitate an evaluation, thus failing to detect tumors earlier^20^.

A Swedish cohort of 604 patients with PSC was followed from 1970 to 1998. The authors found that cancer was the cause of death in 44% of subjects with an increased risk of hepatobiliary malignancy of 161 times^33^. Another study of patients from 1976 to 2000 at the Mayo Clinic in Rochester, Minnesota, found that the approximate risk of CCA in patients with PSC was 0.6% per year, which translates into an increased risk of 1,560 times compared to the general population^22^. A later Mayo cohort from 1995 to 2015 found that 78% of PSC patients developed CCA^120^. In a population-based cohort study, the risk of CCA was 28 times greater in PSC‐IBD patients than in IBD-only patients^113^. It has been well demonstrated that a longer duration of IBD is associated with an increased CCA risk. However, patients treated with colectomy did not have a decreased risk of CCA^121^.

Surveillance programs for PSC-CCA have been trialed using approaches such as checking serum carbohydrate antigen 19-9 (CA 19-9) levels or different imaging modalities. AASLD practice guidance on PSC and CCA recommends annual surveillance with abdominal imaging, preferably by MRI/MRCP with or without serum CA 19‐9 for PSC patients over 18 years of age and with large-duct PSC^122^.

How the milieu of PSC and IBD leads to CCA is unknown. A recent genetic analysis of liver samples from PSC patients with different biliary tract cancers found alterations in genes such as *TP53, KRAS, CDKN2A, SMAD4*, and *HER2/ERBB2*^123^. Interestingly, similar changes in some of these genes are seen in fluke-associated CCA, which suggests a role for chronic inflammation in the development of both kinds of CCA^123,124^.

Importantly, the Greten group demonstrated the functional involvement of the PSC/colitis-dysbiosis-polymorphonuclear myeloid-derived suppressor cell axis in CCA development, not only describing the pathologic connection of the leaky gut and CCA but also highlighting the important aspects of CCA immunotherapies in the clinic^125^.

## Conclusion

PSC-IBD has a distinct genetic background, microbiota composition and clinical features that distinguish it from classical IBD, therefore implying that it is a unique disease entity that is representative of the crosstalk of the gut–liver axis. Although genetics play a limited role in the pathogenesis of PSC, PSC-IBD is classified as an immune-mediated disease. Growing evidence suggests that BAs and the microbiota play important roles in the pathogenesis of PSC-IBD, necessitating further research exploring the interaction of BAs and the microbiota. To date, there are no proven treatments for PSC-IBD. However, an advanced understanding of the pathogenesis of PSC-IBD helps to develop new therapeutic agents. Given the high risk of malignancies associated with PSC-IBD, it is critical to identify patients at high risk and implement appropriate surveillance and monitoring strategies.
